# Experimental data for an air-conditioning system identification

**DOI:** 10.1016/j.dib.2019.104316

**Published:** 2019-07-25

**Authors:** Hermes Ramírez, Javier Jiménez-Cabas, Antonio Bula

**Affiliations:** a Department of Mechanical Engineering, Universidad del Norte, Barranquilla, Colombia; b Departamento de Ciencias de la Computación y Electrónica, Universidad de la Costa, Barranquilla, Colombia

**Keywords:** Mini split, R410A, Design of experiment, Energy, Psychrometry, Refrigeration cycle

## Abstract

In this article, data from a 3.5kW mini split air conditioning is presented. A screening 2^3^ design experiment was performed to evaluate the effects of indoor air temperature, absolute humidity and evaporator fan speed in the performance of the air conditioning. This dataset also includes noise factors as outdoor air temperature, fans energy consumption and response as indoor air exit conditions.

**Table:** Specifications Table

Subject area	*Mechanical Engineering*
More specific subject area	*Refrigeration and air-conditioning*
Type of data	*Excel files*
How data was acquired	*Experimental data obtained from a 3.*5kW *mini-split air-conditioning system using R410A as refrigerant. The instrumentation used was: (10) Digital thermometers Maxim DS18B20 (* ± *0.5°C accuracy)* *(4) Pressure transducers Full Gauge Controls SB69 (* ± *1psig error)* *(3) Humidity sensors Honeywell HIH 4000 (* ± *0.5% error)* *(1) Liquid flow sensor McMillan FLO model 102 (* ± *1.0% error Full Scale)* *(3) Linear current Sensor Allegro ACS712 (1.5% error)*
Data format	*Temperature, flow and relative humidity: Raw.* *Pressure and power consumption: Calculated*
Experimental factors	*Before any experimental test, electrical wires connections were checked.*
Experimental features	*The experimental tests were conducted in a 3.*5kW *mini-split air-conditioning system (see*Fig. 1*). The inlet temperature and inlet humidity of the indoor air, and evaporator fans speed were manipulated. The following variables were measured: outlet temperature and outlet humidity of the indoor air, temperature and pressure of the refrigerant, electrical currents of the compressor and evaporator fans, and inlet and outlet temperatures of the outdoor air.*
Data source location	*Laboratorio de Termofluidos, Universidad del Norte, Barranquilla, Colombia*
Data accessibility	*Data is with this article.*

**Table:** 

**Value of the data** • A detailed database for the steady-state response of a 3.5kW mini-split air-conditioning system in a tropical climate zone. • This unique multivariate experimental design and data collection consider 3 factors that potentially influence the performance of a 3.5kW mini-split air-conditioning system in a tropical climate zone. • The data can be used by other researchers to carry out models validations, by providing a common benchmark. • This data can be used to comparing the performance of the air-conditioning system with the performance obtained in other climate zones.

## 1 Data

This paper presents the experimental data obtained from a 2^3^ screening design experiment. The experiment was performed to evaluate the effects of the inlet temperature and inlet humidity of the indoor air, and evaporator fan speed on the performance of a mini split air-conditioner. The system consists of a 3.5kW cooling capacity mini split with inverter and a seasonal energy efficiency ratio of 17, in this system, 21 sensors were installed in order to measure refrigerant, air, and current data. Data describe the inlet and outlet conditions of indoor and outdoor air (temperature and relative humidity), refrigerant R410A (temperature, pressure, volumetric flow) and power consumption (current) of the equipment, Table 1 shows the description and location of each sensor. Each run was performed at steady state operation for 1 hour and 45 minutes. To assure steady state, measurements started 1 hour after the air conditioning startup or change in the air inlet conditions as recommended in ASHRAE/AHRI 210 [1] and ISO 5151 standards [2].

**Table 1:** Instrument location and reference.

Reference	Description	Precision/Accuracy
T1	Refrigerant temperature at compressor inlet	±0.5 °C
T2	Surface temperature of the compressor	±0.5 °C
T3	Refrigerant temperature at compressor outlet	±0.5 °C
T4	Outdoor air temperature at condenser inlet	±0.5 °C
T5	Outdoor air temperature at the condenser outlet	±0.5 °C
T6	Refrigerant temperature at condenser	±0.5 °C
T7	Refrigerant temperature at condenser outlet	±0.5 °C
T8	Surface temperature of the Capillary tube	±0.5 °C
T9	Indoor air temperature at the evaporator inlet	±0.5 °C
T10	Indoor air temperature at the evaporator outlet	±0.5 °C
P1	Refrigerant pressure at compressor inlet	±1 PSIg
P2	Refrigerant pressure at compressor outlet	±1 PSIg
P3	Refrigerant pressure at condenser outlet	±1 PSIg
P4	Refrigerant pressure at DX outlet	±1 PSIg
H1	Indoor air relative humidity at air hood	±0.5% error
H2	Indoor air relative humidity at the evaporator outlet	±0.5% error
H3	Indoor air relative humidity at the evaporator inlet	±0.5% error
I1	Compressor current consumption	1.5% error
I2	Condenser fan current consumption	1.5% error
I3	Evaporator fan current consumption	1.5% error
F1	Refrigerant liquid flowmeter at condenser outlet	±1.0% error Full Scale

## 2 Experimental design, materials and methods

**Fig. 1:**
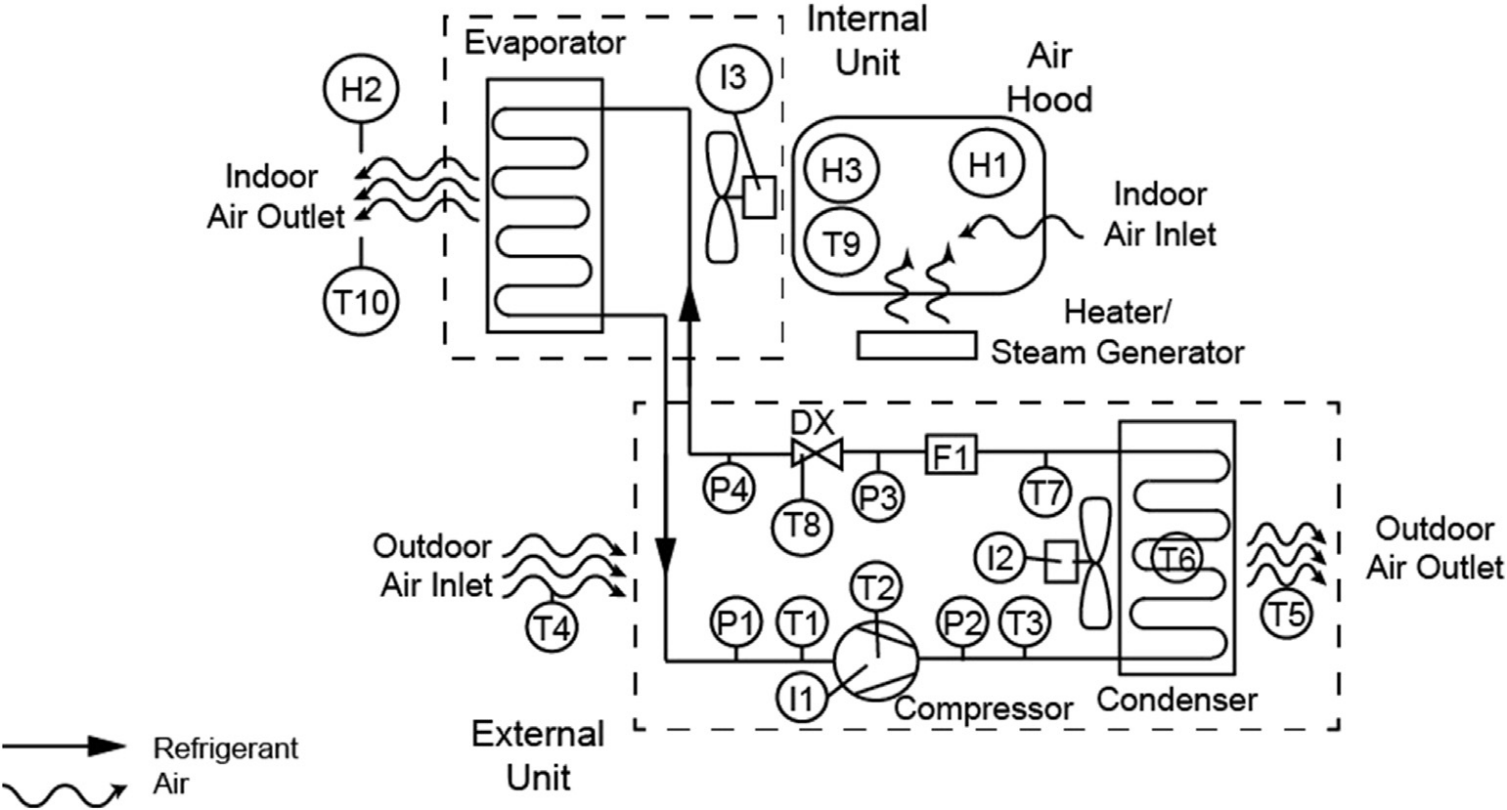
Test rig experiment scheme.

Fig. 1 shows the experimental test rig scheme; it is localized in the Universidad del Norte, Barranquilla Colombia. Barranquilla is a tropical climate city, whose mean annual outdoor air temperature is in the range from 24 °C to 30.1 °C; besides, the annual mean outdoor air relative humidity is in the range from 80 to 85%. To assure indoor air inlet conditions in the evaporator, an air hood was installed at evaporator air inlet; using an electrical heater, and/or a steam generator (depending on the situation) to maintain the inlet conditions at the accepted range during the time measurements. Table 1 shows the description of each sensor installed in the test rig.

Considering that this is not a standard test, the accuracy of each sensor is accepted as recommended by the US Department of Engineering [3], temperature of indoor air at the evaporator inlet was stablished in 23 °C and 28 °C with ±1 °C as an acceptable range, which represents the typical range of indoor air temperature in rooms and offices. In the other hand, the absolute humidity is not directly measured; hence, relative humidity is adapted to reach the required values. Nevertheless, the relative humidity also depends on the air temperature, so to reach the 2 absolute humidity accepted values, 4 values of relative humidity are required, in Table 2 the values are presented.

**Table 2:** Indoor air inlet evaporator humidity levels.

Absolute humidity level (kg/kg)	Relative humidity level (%)	Indoor air inlet temperature (°C)
0.010	62	23
0.010	46	28
0.014	75	23
0.014	55	28

The evaporator fan has 3 velocity levels and 1 auto mode; during the experimental tests, the evaporator fan speed was recorded as categorical data. Actual air speed was not measured; however, this factor was considered as it might influence results (i.e. outlet air temperature at evaporator). Two (2) Fan speed levels were chosen in this experiment (low-level speed and high-level speed). Based on Table 2 data, a fan level, indoor air temperature, and indoor air relative humidity level experimental design was performed; to assure homogeneity in the results, the running order for the test were randomized.

Dataset is stored in an Excel file, with 16 worksheets, one for each experimental test. The first column for each worksheet shows the time elapsed between the first and the given measured data; date is not included in the data because the acquisition unit doesn't capture the date time. The data for all the 21 measurements are presented resulting in 10700 store data, a mean of 6700 data per run. The absolute pressure data was calculated by the addition of the gauge pressure measurement from the experimental system and the atmospheric pressure (at sea level = 14.7 PSIa).

Calculated power consumption is the product of current data measure from the experimental system by voltage (120V). Finally, Table 3 shows the order of the running test and the indoor air inlet conditions for each test.

**Table 3:** Experiment running order.

Run	Control Parameters		
	Fan Speed Level	Indoor air inlet conditions	
		Temperature (°C)	Relative Humidity (%)
1	Level 1	28	46
2	Level 3	28	55
3	Level 1	23	62
4	Level 3	23	75
5	Level 3	28	46
6	Level 3	23	62
7	Level 1	23	75
8	Level 1	28	55
9	Level 1	28	46
10	Level 3	28	55
11	Level 1	23	62
12	Level 3	23	75
13	Level 3	28	46
14	Level 3	23	62
15	Level 1	23	75
16	Level 1	28	55
